# A Pneumatic Tactile Sensor for Co-Operative Robots

**DOI:** 10.3390/s17112592

**Published:** 2017-11-10

**Authors:** Daoxiong Gong, Rui He, Jianjun Yu, Guoyu Zuo

**Affiliations:** 1 Faculty of Information Technology, Beijing University of Technology, Beijing 100124, China; herui@emails.bjut.edu.cn (R.H.); yujianjun@bjut.edu.cn (J.Y.); zuoguoyu@bjut.edu.cn (G.Z.); 2 Beijing Key Lab of the Computational Intelligence and Intelligent System, Beijing 100124, China

**Keywords:** force sensor, pneumatic tactile sensor, linearity, repeatability, hysteresis

## Abstract

Tactile sensors of comprehensive functions are urgently needed for the advanced robot to co-exist and co-operate with human beings. Pneumatic tactile sensors based on air bladder possess some noticeable advantages for human-robot interaction application. In this paper, we construct a pneumatic tactile sensor and apply it on the fingertip of robot hand to realize the sensing of force, vibration and slippage via the change of the pressure of the air bladder, and we utilize the sensor to perceive the object’s features such as softness and roughness. The pneumatic tactile sensor has good linearity, repeatability and low hysteresis and both its size and sensing range can be customized by using different material as well as different thicknesses of the air bladder. It is also simple and cheap to fabricate. Therefore, the pneumatic tactile sensor is suitable for the application of co-operative robots and can be widely utilized to improve the performance of service robots. We can apply it to the fingertip of the robot to endow the robotic hand with the ability to co-operate with humans and handle the fragile objects because of the inherent compliance of the air bladder.

## 1. Introduction

With the development of robotics, more emphasis has been placed on the application of robots co-operating with human beings to fulfil certain tasks. The ability of “human-like touch” is eagerly anticipated for co-operative robots and the tactile sensor will thus find a favorable ground in human-robot co-operative applications. In these applications, instead of performing routine, preplanned tasks in structured industrial environments [1], robots featuring with dexterous grippers and manipulators and operating in the unstructured daily living environments of people, to co-operate and interact with people. 

A co-operative robots should be able to sense multiple tactile information to realize the human-like touch [1]. A human-like touch may include: (1) the sense of the contact force, which is very important for co-operative robots to perform force control, stable grasp and in-hand manipulation [2,3]. (2) the sense of the textures, deformation and elasticity of the touched object’s surface, which is an important capability for a service, autonomous or assistive robot to distinguish between human and objects [4,5] and thus adopt a corresponding control strategy according to the properties of the objects and tasks. (3) the sense of slippage and vibration of the grasped object, which is important in robotic dexterous manipulation because it provides useful information for grip force adjustment and stable grasp control [3,6,7,8,9]. Additionally, the tactile sensor itself should be compliant enough to yield to unexpected perturbation of contact force when it collides with obstacles (or humans), so that it can guarantee the safety of the robot system itself, the human and the environment [4]. 

A human-like compliant touch is still a major challenge yet solved in the robotics community and its realization relies on the development of a tactile sensor. Many kinds of tactile sensors have been invented based on capacitive, inductive, conductive, piezoresistive, piezoelectric, optical, magnetic, and barometric principles etc., and there are several review papers to provide good information on them [2,4,6,8,10,11,12]. Every kind of tactile sensor has its own advantages and disadvantages in different applications. For example, Ribeiro et al. studied a miniaturized light force sensor for the detection of small forces [13]. Fujiwara studied a tactile sensor based on optical fiber specklegram for high sensitivity and spatial precision of tactile sensing [14]. De Maria et al. proposed an elastically deformable sensor with LED-phototransistor couples and a signal-discrepancy-based slipping detection algorithm for stable grasping [15]. However, optical tactile sensors may have a bulky and complicated system structure and be expensive and the magnetic sensors’ response may be vulnerable to external magnetic change [16]. Thus, it is important for a tactile sensor robust, flexible and compliant to be deployed on a robotic finger to interact with human and environment safely and effectively [17].

In order to realize soft, compliant force sensing, a lot of force and tactile sensors are developed with flexible material and/or a liquid medium [11,18]. Yu et al. utilized four small-sized soft piezoelectric tactile sensors to derive the three-dimensional force components [19]. Lee et al. studied tactile sensors with the sensing area of 2 mm width [20]. Oddo et al. proposed a piezoresistor based micro-sensor array and packaged it with polyurethane for the compliance and softness of the human finger pad [21]. Dobrzynska et al. studied a flexible force sensor based on polyimide [22]. Chun et al. developed a tactile sensor with microstructures inspired by human fingerprints using single layer graphene for surface texture recognition [23]. Wong et al. studied a flexible artificial skin based on microfluid [24]. Vogt et al. developed soft multi-axis force sensors using elastomer and liquid metal [25]. Yeo et al. developed a triple-state liquid-based resistive microfluidic tactile sensor with high flexibility, durability and sensitivity [26]. Chen et al. developed a capacitive pressure tactile sensor with carbon nanotubes dispersed in liquid crystal and realized an adjustable measurement range via changing the driving frequency and voltage [27]. 

A pneumatic sensor with soft air bladders can obtain force and tactile information via sensing the pneumatic pressure. Thus, many people utilize it as a soft force measurement system. Choi et al. studied a soft three-axis force sensor with radially symmetric pneumatic chambers [28]. Kong et al. developed insole sensor using silicone tube and pressure sensors for wearable gait analysis application [29]. Abinaya et al. also studied an air pressure sensor based smart shoes for gait monitoring and reported that the pressure changes in the air bladder are linearly proportional to the exerted force [30]. Nozawa et al. proposed a method of sensing pneumatic pressure in air cushions for human-robot cooperative heavy object manipulation [31]. The air bladder as a sensing device possesses some noticeable advantages for co-operative applications: the air in the bladder is compressible, thus the air bladder is compliant and makes soft contact between the human and the robot. This property is very important for the safety and the friendly human-robot interaction. Besides, user can customize the size, shape and air volume inside the air bladder according to the requirement of robotic application. Therefore, it is worth studying the pneumatic tactile sensor with air bladder.

Although these tactile sensors are soft and compliant, most of these sensors are limited in human-robot interaction application due to their small measurement range or area of the sensitive part, as well as high manufacturing complexity and cost. Inspired by mechanoreceptors in humans’ glabrous skin, some researchers try to integrate different sensors into a multimodal one to detect more than one kind of sensing information [32,33,34]. The multimodal sensor requires high-density integration technology with many kinds of (e.g., force and vibration) sensors as well as a delicate layered organization of the devices and their functional relationships [34] and may increase the structural complexity of the sensor and restrict the behaviors of the gripper. Therefore, some researchers seek a way to fabricate a tactile sensor with the finger-shape so that they can apply the sensor to the robots easily. Yang et al. studied a novel pneumatic soft sensor for measuring the contact force and the curvature of a soft gripper [35]. The sensor is composed of a sensing body and a pressure sensor, and endows the finger a self-sensing ability. Yamazaki et al. studied a finger-shaped end effector embedded with mechanoreceptor [36]. Hosoda et al. proposed a finger-shaped robotic end effector with randomly implanted strain gauges and polyvinylidene fluoride films to imitate the organization of human fingers [37]. Jamone et al. proposed an anthropomorphic robotic hand contains magnet and Hall Effect sensor inside the silicone fingertip for the sense of force [16]. Sato et al. studied a finger-shaped vision-based sensor—GelForce—to detect multi-axis force distribution on the finger surface with CCD camera [38]. The BioTac sensor is a finger-shaped tactile device that has a bone-like core and an elastomeric skin filled with conductive liquid to sense temperature, contact force and micro-vibrations [33]. Koiva et al. produced a tactile fingertip sensor for the Shadow Robot Hand that incorporates tactile sensors with signal acquisition electronics [39]. Nishimura et al. presented a robotic hand with fluid fingertips that can stably grasp a variety of soft and fragile objects such as tofu [40]. 

Therefore, it is necessary to find a sensor that has a simple structure and manufacturing process, high accuracy, good compliance and easy integration with a gripper. We propose a prototype of pneumatic tactile sensor with the shape of the pad of the finger for co-operative robotics and validate its performance via experiments in this paper. This paper is organized as follows: the construction of the pneumatic tactile sensor is illuminated in Section 2. Experiment results are presented in Section 3 to validate the pneumatic tactile sensor’s performance of force, texture, and vibration sensing. The advantages and some potential applications of the pneumatic tactile sensor are summarized in Section 4.

## 2. Materials and Fabrication

The pneumatic tactile sensor is composed of an air bladder and a signal processing circuit. The air bladder is a section of latex tube with an inside diameter of 9 mm, outside diameter of 12 mm and length of 70 mm. We take a plastic tube plug (PP-10) with diameter 10 mm to seal one end of the latex tube with cyanoacrylate super glue and use a plastic quick coupling (PU-04) to seal another end with the glue and connect the PU-04 to the pressure sensor via pipe. The size of the working part of the air bladder is an analogue of the pad (distal phalange) of the index finger of human with almost the same width and double length. The bladder deforms when we apply a force on it and the pressure in the bladder changes at the same time. This air bladder can endure a maximum inner air pressure of 150 kpa, which is 50 kpa higher than the atmospheric pressure. 

**Figure 1:**
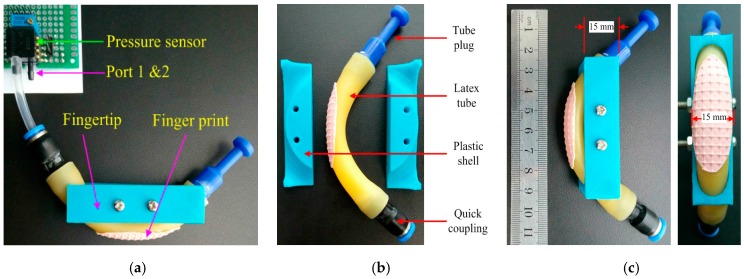
The pneumatic tactile sensor. (**a**) the configuration of the sensor with the fingertip connected to the MPXV5050DP; (**b**) the components of the fingertip; (**c**) the size of the fingertip.

We implant a pressure difference sensor MPXV5050DP of Freescale Semiconductor [41] in the air bladder to detect the change of pressure. MPXV5050DP has a sensing range of 0–50 KPa between its two ports, as shown in Figure 1, one port is open to the atmosphere and another port is connected to the air bladder, so it can measure the difference of pressure in the bladder caused by the force applied on the air bladder. We replug the pipe into PU-04 every time we use the pneumatic tactile sensor and thus the latex tube is filled with air of the same pressure of the atmosphere no matter what the temperature of the environment is. If we neglect the atmospheric pressure variation caused by the change of temperature, then we can ignore the effects of temperature on the measured signal. The output of the pressure difference sensor is transferred to a data acquisition system (DAS) based on ARM STM32 Microprocessor. The processor samples the output of the pressure sensor with A/D module and then performs the processing of Kalman Filtering and Discrete Fourier Transform (DFT), with consideration of the nature of the tactile signal and finally transmits the result to PC via the Universal Synchronous Asynchronous Receiver Transmitter (USART) for tactile sensing.

The pneumatic tactile sensor entity is shown in Figure 1. A plastic shell made by 3D print is adopted to fix the air bladder and serve as a fingertip of a robotic hand. From Figure 1, we can learn that the pneumatic tactile sensor entity is easy to fabricate, lightweight, cheap and low-power consumption. The total weight of the pneumatic tactile sensor entity is 25 g and the cost is 80 RMB (including the price of the 60 RMB for MPXV5050 pressure sensor) and the power consumption of 0.053 W for MPXV5050 according to its datasheets. 

**Figure 2:**
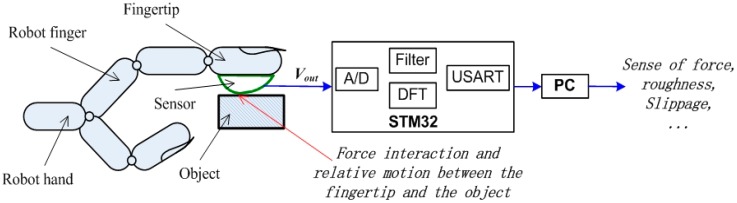
The diagram of the pneumatic sensor’s construction and applications.

A diagram is presented in Figure 2 to show the sensor be attached on a robotic hand to obtain the tactile information (i.e., the force, the roughness of the touched surface, the slippage of the grasped object and etc.). The pressure difference sensor has two input ports, one of which detects the pressure in the air bladder and another input port always detects the pressure of atmosphere, therefore we can record and detect the change of pressure in the bladder and further obtain the tactile information we need.

According to the Datasheet of MPXV5050 [41], the output of the pressure difference sensor can be formulated as:

(1) $$V_{out}=5\times (0.018\times \Delta p+0.04)\pm 0.1125$$

where $\Delta p=p_{1}-p_{2}$ is the difference between the pressure in the air bladder $p_{1}$ and the pressure of the atmosphere $p_{2}\approx 101\;\mathrm{kPa}$. The constant 0.1125 is a computing result with taking both the temperature error and the pressure error into consideration, i.e., for the temperature within the range of 0–85 °C and the pressure difference of the two ports of MPXV5050DP within the range of 0–50 kpa. Obviously, the studied sensor covers the application condition of a co-operative robots in terms of both the pressure sensing range and the environment temperature range.

## 3. Parameters and Performance

We apply the sensor to the gripper of a robot manipulator to serve as the fingertip, which can sense the force exerted on it, the surface of the object it touched and the relative sliding between the grasped object and the robotic hand. We validate the parameters and performance of the tactile sensor via experiments with a sampling period of 0.8 ms, which is the fastest sampling speed our system can achieve and also a sufficient for the environment sensing application of a robotic hand. Taking human performance as a reference, the normal neuromuscular delay of slip detection is 74 ± 9 ms. Therefore, the sampling period is adequate for providing sufficient reaction time for the sensing and control of a robot hand [33].

### 3.1. Sensing of Force

#### 3.1.1. Linearity

**Figure 3:**
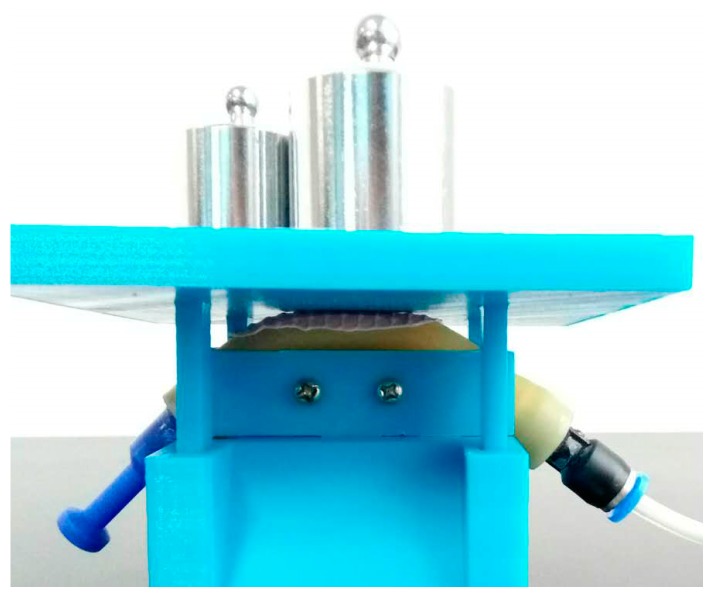
The experiment of force sensing.

**Figure 4:**
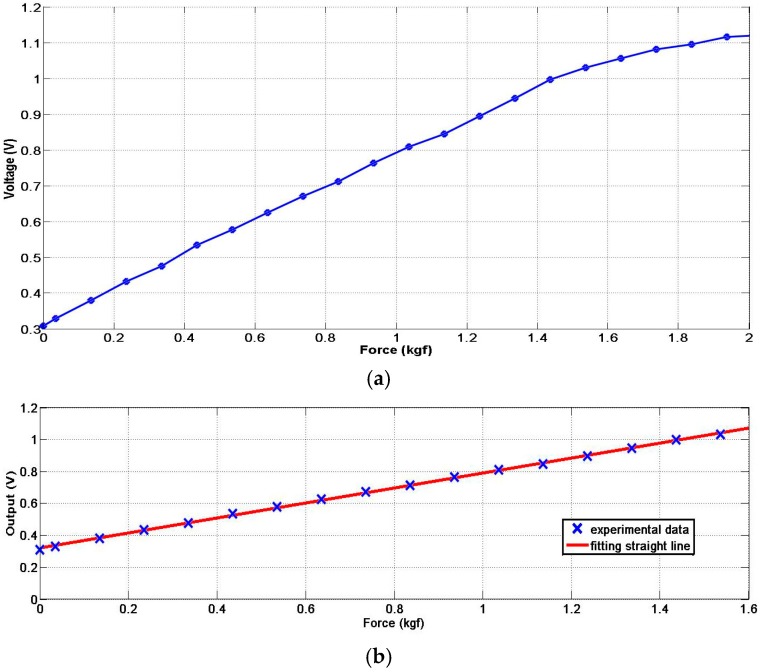

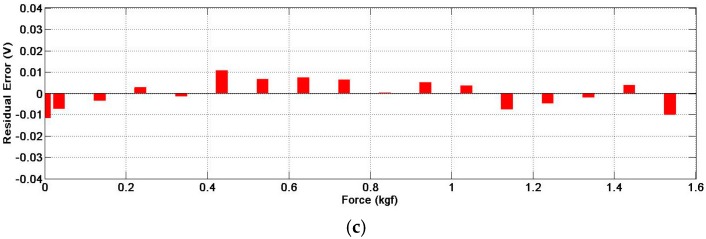
The force-voltage curve, the fitting line and the residual error of force sensing. (**a**) the force-voltage curve; (**b**) the fitting line in the range of good linearity; (**c**) the residual error.

When we exert a force on the air bladder, the pressure in the bladder increases. The pressure difference sensor detects the change of pressure and thus can measure the exerted force. In order to get the force-voltage curve of the tactile sensor, we conduct an experiment as shown in Figure 3: we put different counterweights on a tray, which has four slide guides to ensure its vertical motion so that the weight will totally exert on the pneumatic sensor. We recorded every pair of the total weight put on the sensor and the output voltage of the sensor and this produced the curve presented in Figure 4a. The curve has good linearity in the range of 0–1.6 kgf, and then the growth trend of the curve will slow down and finally become a horizontal line at 2 kgf because the air bladder is totally pressed flat that point.

We add the weight to the sensor with an increment of 0.1 kg. The cross mark in Figure 4b indicates the force exerted on the sensor, in which the left-most one means no external force is exerted and 0.321 is thus the offset of Formula (2). The second cross mark on the left side indicates the force caused by the weight of the tray (0.035 kg) and the following cross marks indicate the force caused by both the corresponding counterweight and the weight of the tray.

We apply the least-square method for data fitting in the range of 0–1.6 kgf on the raw data of Figure 4a and we get the linear input-output relationship as well as the residual error of the pneumatic sensor as shown in Figure 4b,c. From Figure 4, we can learn that the sensor has good input-output linearity in the range of 0–1.6 kgf for force sensing and we can formulate the fitting line of the pneumatic sensor as the following function:

(2) $$V_{o}=0.468\times W+0.321$$

where W (kgf) is the force (weight) exerted on the sensor and 0.321 is the offset caused by the initial pressure of the compressed air in the bladder.

#### 3.1.2. Repeatability

**Figure 5:**
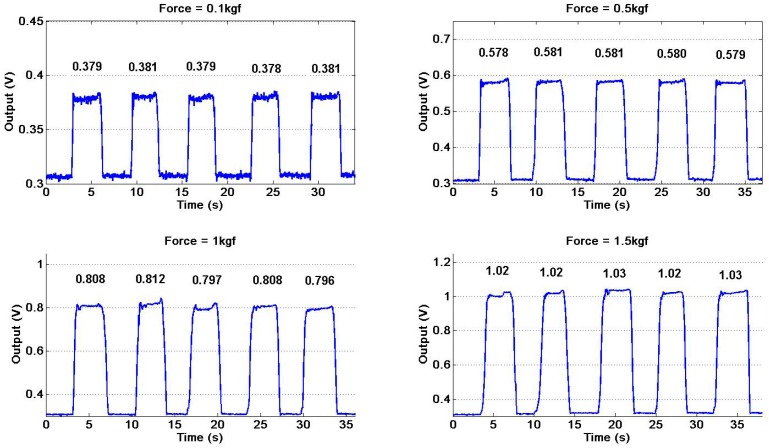
The repeatability of the sensor under different forces: 0.1 kgf, 0.5 kgf, 1.0 kgf and 1.5 kgf.

We define the repeatability as the variation of the sensor reading out of several experiments under the same conditions. In order to verify the repeatability of the pneumatic sensor for force sensing, we adopt the experimental setup of Figure 3 and repetitively apply-remove a fixed force by putting-and-removing the counterweights of 0.1 kg, 0.5 kg, 1.0 kg and 1.5 kg on the pneumatic sensor, respectively. Figure 5 shows the experimental results and the numbers in the figure represent the average values of steady signals, which are expected to be the same for each experiment. From Figure 5, we can learn that the variation of sensor reading is very small for the same exerted force and the pneumatic sensor shows a good repeatability of force sensing. 

In addition, from Figure 5 we can learn that the values of rising time are 200, 300, 400 and 500 ms for the force of 0.1, 0.5, 1.0 and 1.5 kgf respectively. Note that the pneumatic sensor has good response time and the higher contact force of the sensor, the longer rising time. Because the sensor is compliant and it has a bigger deformation when we exert a higher force on it. The compliant feature may bring some advantages to the sensor: The softness and flexibility of the air bladder is approximate to those of the human muscle and it may make the robot hand more friendly and comfortable when directly interact with human as a service robot or handle some fragile articles.

#### 3.1.3. Hysteresis

**Figure 6:**
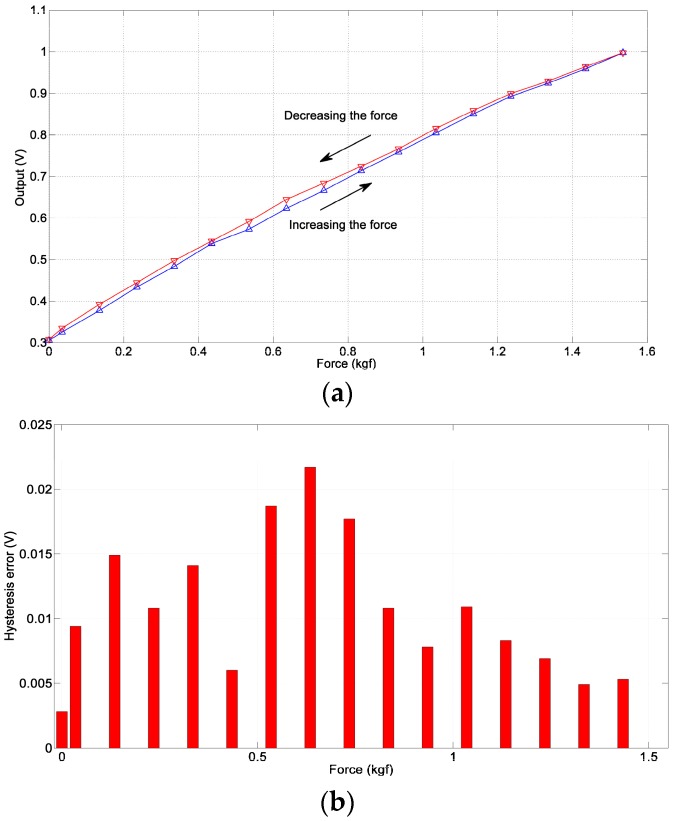
The hysteresis of the sensor. (**a**) The hysteresis curve; (**b**) The hysteresis error.

We define hysteresis as the maximum difference between the two output values corresponding to the same input obtained on different trajectories of the sensor. We test the hysteresis characteristics of the sensor by gradually increasing the force applied on the sensor from 0 to the maximum of linearity range (i.e., 1.5 kgf) and then gradually decreasing the force to 0. The experiment result is presented in Figure 6. It validates that the pneumatic sensor has a very small hysteresis error, which is good enough for the co-operative application of robotics because it will not cause big sensing error no matter in the course of increasing the force or in the course of decreasing force.

### 3.2. Sensing of Surface Characteristics

In robotic applications, the tactile sensor must be able to characterize different textures of the surface it touched, including the coarseness/roughness of the surface and the softness/stiffness of the object. 

#### 3.2.1. Sensing of Roughness

**Figure 7:**
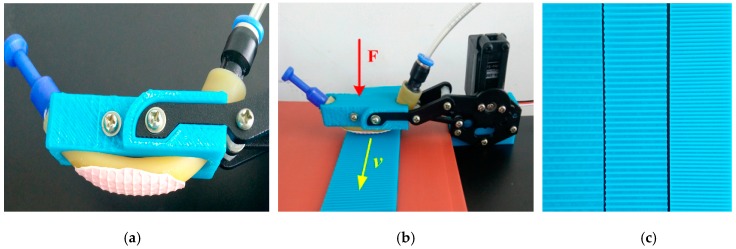
The experiment of sensing the surface of object. (**a**) a fingertip with the fingerprint; (**b**) the relative movement between the fingertip and the test surface; (**c**) the test grooves.

Object recognition is an important requirement in human-robot interaction and autonomous manipulation [10]. Tactile sensors can provide information about local surface texture of the object via the relative movement between the fingertip and the surface of an object in a manner of “action for perception” [12], because vibrations will take place during slip due to the roughness of the surfaces in contact. Ridged surfaces (grating patterns) with grooves and ridges have been used for experimental analysis of the roughness sensing tactile sensor and experiment results may be classified as a successful attempt to achieve roughness sensing in the case of medium-coarse surface [21,34]. In order to test the sensor’s ability of detecting the roughness of the surface it touched, we make the 3D printed test surface with ridges and grooves of equal width (4, 3 and 2 mm, respectively, the right of Figure 7). We press the sensor on these test surfaces with the same force of 0.056 kgf and pull the surfaces with approximately the same velocity, namely to pull a distance of 150 mm within 2.13, 2.34 and 2.20 s, respectively (the middle of Figure 7). Since the roughness is detected by vibration, i.e., the subtle pressure changes due to surface roughness during the relative slip motion between the sensor element and the touched object, we attach a fingerprint on the sensor to enhance the vibration for roughness sensing (the left of Figure 7).

**Figure 8:**
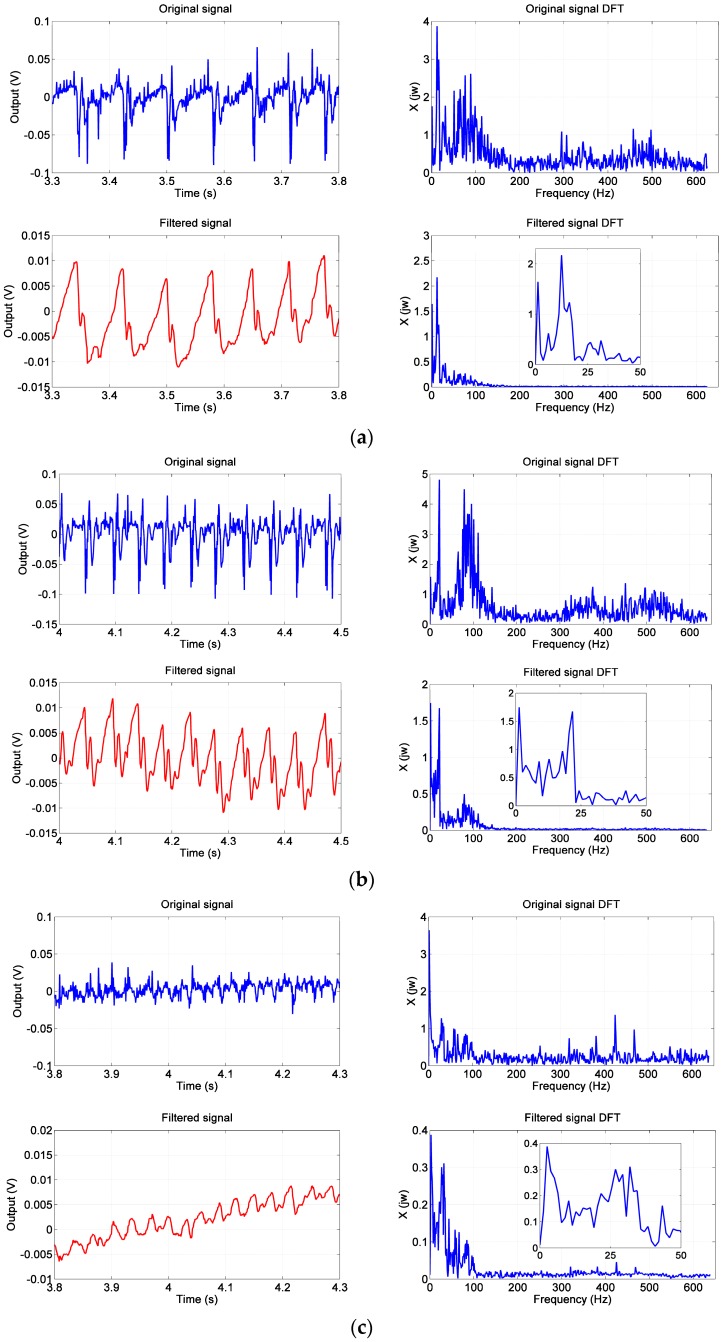
The results of surface with 4, 3 and 2 mm wide ridges, including the raw data, the result of Kalman filtering, the result of DFT and the result of both Kalman filtering and DFT. (**a**) the result of the surface with 4 mm wide ridges; (**b**) the result of the surface with 3 mm wide ridges; (**c**) the result of the surface with 2 mm wide ridges.

Figure 8 presents the experiment results of surface with different ridge widths. Kalman filtering and DFT are applied to the experiment data for noise elimination and frequency analysis, respectively. Different vibration frequency means different roughness of the contact surfaces. From the curves of Kalman filtering we can learn that the rougher (wider ridges and grooves) the surface, the lower the frequency of the output signal. The DFT analysis reveals that the frequencies corresponding to the ridge width of 4, 3 and 2mm are 12, 20 and 30 Hz, respectively, thus the pneumatic sensor can detect the roughness of the touched surface.

The detectable vibration frequencies of human hand vary from 5 to 50 Hz [10] and a frequency response of 20–60 Hz is believed to be adequate for social Human-Robot Interaction [4], thus the pneumatic sensor is fit for the application of robot collaborating with human.

#### 3.2.2. Sensing of Softness

**Figure 9:**
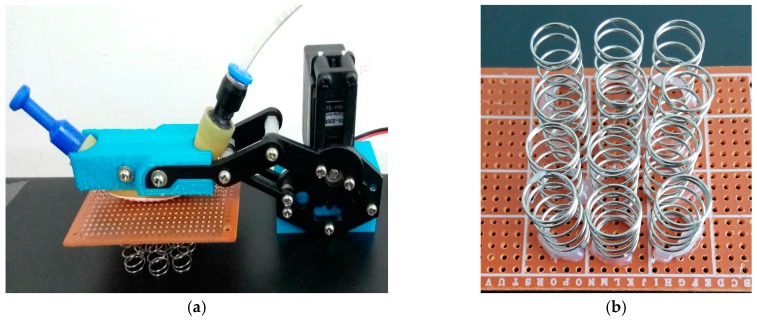
The experiment of detecting softness. (**a**) the experiment; (**b**) the softness simulated by springs.

Features of softness and deformability of the object are crucial for the robot performing manipulation tasks. The softness/stiffness of the object can be detected via the variation of both the force and the joint angle. In order to test the ability of sensing the softness/stiffness of the object it touched, we designed an experiment as shown in Figure 9. We place some soft springs parallel under a flat plate and take the robot finger pressing the plate to detect the softness of the board-spring system. The more springs we placed under the plat, the higher stiffness the system is. 

**Figure 10:**
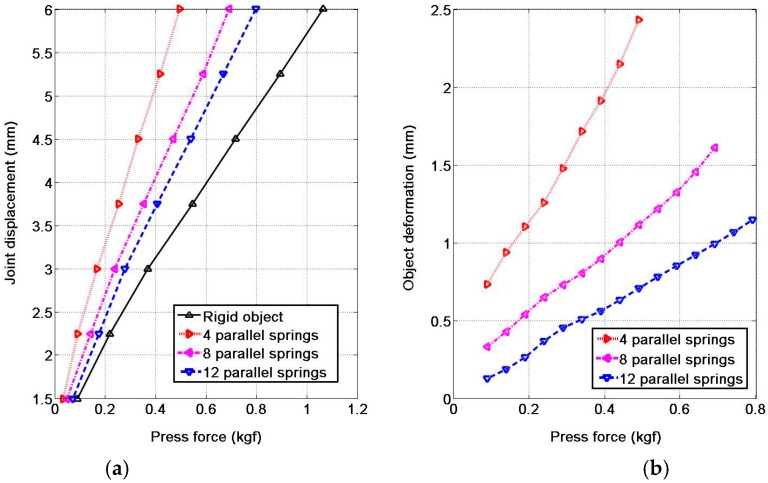
The experiment result of softness. (**a**) the raw data; (**b**) the curve of softness.

In Figure 10a, we present the raw data of the sensor pressed on the board-spring system with 0, 4, 8 and 12 springs, respectively. In this experiment, we define the joint displacement of the robotic finger as zero when the fingertip just begins to touch on the plate and control the motion of the robotic fingertip with a displacement increment of 0.75 mm for every data recording (Figure 10a). The joint displacement includes two parts: (1) the deformation of the pneumatic sensor itself; (2) the deformation of the springs. The deformation of the sensor is exactly the joint displacement when the finger presses on a rigid body (the zero spring deployment) and the deformation of the springs is exactly the difference between the output of pressing hard surface and the output of pressing soft surfaces. Therefore, we can obtain the curve of softness of the experiment objects via shape-preserving interpolation on the empirical curve of Figure 10a and we get the result as shown in Figure 10b. From top down are the force-deformation curves of 4, 8 and 12 springs, respectively and the slope characterizes the softness of the experiment objects, i.e., 4.1, 2.0 and 1.4 mm/kgf, respectively.

This experiment indicates that the pneumatic sensor can perceive and distinguish the different softness/stiffness of the object and thus can provide information for the robot to handle different objects, for example, the rigid object like glass cup, the elastic object like ball and the soft object like sponge.

### 3.3. Sensing of Slippage

**Figure 11:**
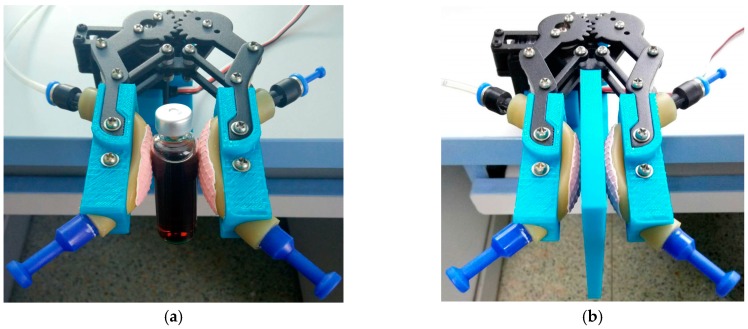
The experiment setup of sensing slippage. (**a**) clamping an object with smooth surface; (**b**) clamping an object with rough surface.

Detection of slippage plays an important role in grip force control for robotic hands to achieve a stable grasp as well as other manipulation tasks [12,42]. A robot hand experiences mechanical vibrations during slippage. Therefore, people usually take vibrations as the slip-signals [10,42]. We setup an experiment as shown in Figure 11 to test the ability of sensing slippage of the pneumatic tactile sensor. We clamp objects with smooth and rough surface between the robotic fingertips with pneumatic sensor, respectively and then pull the objects along the plane between the two fingers to make the objects having a potential of slippage and slipping between the fingers, respectively. In order to enlarge the vibration signal, we attached a fingerprint to the robotic fingertip as shown in Figure 11a.

**Figure 12:**
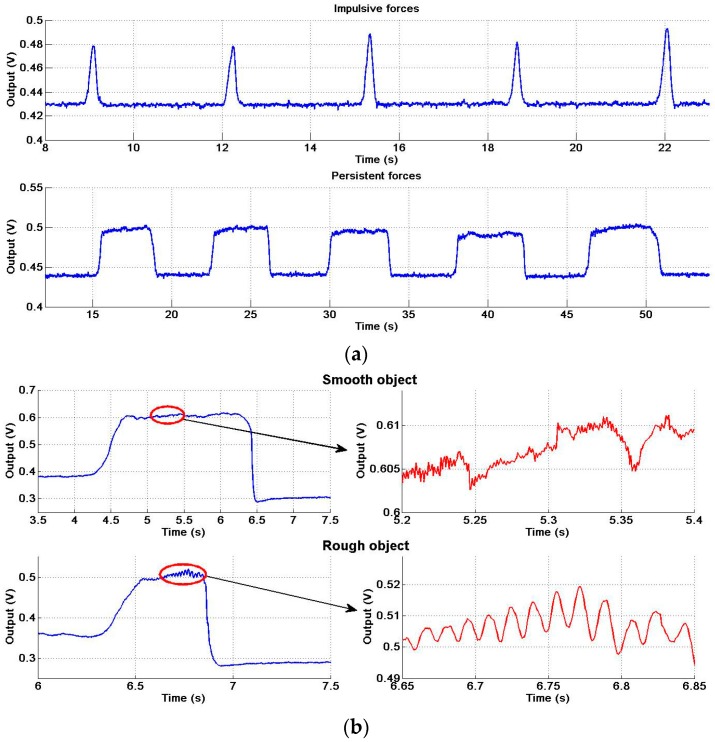
The experiment results of sensing slippage. (**a**) the objects with tendency of slip under impulsive and persistent force; (**b**) the smooth and rough object slipping.

Figure 12a presents the experiment results of the objects with the tendency of slipping under impulsive and persistent forces, respectively. When we exert these forces vertically downward on the objects in Figure 11, it will produce an additional twist deformation to the air bladder, which will result in an increase of the air pressure in the bladder. Thus the pneumatic sensor detects a pulse force and a persistent force as shown in Figure 12a though the force is not exerted on the pneumatic sensor in the normal direction.

Figure 12b presents the experiment results of the objects slipping between the fingers. The force of sliding friction equals to the maximum static friction force. Therefore, the curves in Figure 12b are similar to those in Figure 12a. The sliding brings vibration, so we find a vibration of fixed frequency as shown in the bottom-right part of Figure 12b. The frequency has relevance to the sliding speed and the roughness of the surface of the object as discussed in Section 3.2.1. As shown in both the right part of Figure 12b and the Section 3.2.1, the more smooth the surface of the sliding object, the higher frequency of the vibration signal. Previous work has shown that slip has a range of vibration frequency between 10 and 30 Hz [11]. The pneumatic tactile sensor has appropriate bandwidth to detect the vibration frequencies and thus it can detect the slippage of an object when consider both the change of force and the vibration signal and thus can help the robot to grip object steadily. 

The property of sensing the slip tendency as shown in Figure 12a is noticeable. Most of the tactile sensors are common to normal force transduction, while our sensor shows the ability of sensing both the normal force and the tangential force. Besides estimating the stability of the grasp, this property has an important application in the co-operative robots. When we use a robot to collaborate with a person, for example, the person wants to carry a table away with the help of a robot. Usually the person is the master and the robot is the slave in this situation and the robot with the pneumatic sensor on its hand is able to perceive the motion intention of the person via sensing the motion tendency of the table and thus robot is able to collaborate with the person successfully. Also, with the force and vibration information, the sensor may provide the possibility of achieving the human-like manipulation as shown in [43], i.e., the robots are able to keep objects subjected to load perturbations by adjusting grasping force to prevent slippage and at the same time avoid to cause deformation or damage to the object as human do.

## 4. Conclusions

We fabricated a preliminary prototype of pneumatic tactile sensor and performed a proof-of- principle study in this paper. The developed sensor is simple in structure and easy to fabricate and has been validated capable of detecting a wide range of tactile information via calibration experiments. It not only can achieve good performance in sensing force, vibration and slippage but also can sense the softness of the object and the roughness of the object surface in haptic application. The pneumatic tactile sensor possesses a wide range of sensing ability, good linearity, repeatability and low hysteresis and the sensor also has characteristics of low cost, flexible, lightweight, low energy consumption, simple structure, good compliance and easy to manufacture. Besides these functional performances, the pneumatic sensor also possesses some special features that are very important for its application in robotics. The sensor has inherent human-like compliance because of its mediator of compressible air. Furthermore, the sensor can directly act as the pad of the finger for physical interacting with humans and the environment and it does not need the complicate procedure of installing on a robotic fingertip like an artificial skin or a rigid silicon-based MEMS tactile sensor.

People can customize the size (width, length and thickness), the durability under working loads and the sensing range of the sensor for task-centered design by using different material and thickness of the air bladder. The thicker the wall of the latex tube, the higher air pressure the tube can endure before it bursts and thus larger measuring range of force of the pneumatic tactile sensor. Similarly, the thicker the inner cylinder of the latex tube, the larger applied force the tube can endure before it is pressed flat. The air bladder is flexible for bending and stretching, thus, it has good adaptability for different mechanical structure and is suit for assembling to variable position on the robot. Based on the results of this paper, we will study the fabrication of a pneumatic tactile sensor with the shape of the fingertip and apply it to an anthropomorphic robotic hand in our future research.

Although the pneumatic sensor provides many good properties, it measures only a lumped force acting on the chamber because it measures the pressure change in the air bladder. It cannot provide sufficient tactile information in the situation of spatial resolution is concerned and we may apply some array-based technology to the surface of the air bladder to improve its performance. 

When we apply the sensor to the fingertip of the robot, the intrinsic compliance of the pneumatic sensor will bring the robot hand some feature of human-muscle-like soft touch and thus endows the robot hand the ability of co-operation with human and handling the fragile/deformable object because of the inherent compliance of the air bladder. These properties of human-like compliance, robustness, appearance and tactile sensation will make the co-operative robots more intimate and friendly to the person interacting with it. Therefore, we suggest that the pneumatic tactile sensor is suit for the application of co-operative robots and can be widely utilized to improve the performance of service robots.
